# Axonal protection by combination of ripasudil and brimonidine with upregulation of p-AMPK in TNF-induced optic nerve degeneration

**DOI:** 10.1007/s10792-024-03095-9

**Published:** 2024-04-10

**Authors:** Mizuki Otsubo, Kana Sase, Chihiro Tsukahara, Naoki Fujita, Ibuki Arizono, Naoto Tokuda, Yasushi Kitaoka

**Affiliations:** 1 Department of Molecular Neuroscience, St. Marianna University Graduate School of Medicine, 2-16-1 Sugao, Miyamae-Ku, Kaswasaki, Kanagawa 216-8511 Japan; 2 Department of Ophthalmology, St. Marianna University School of Medicine, Kaswasaki, Japan

**Keywords:** Ripasudi, Brimonidine, AMPK, Glaucoma

## Abstract

**Purpose:**

The ROCK inhibitor ripasudil hydrochloride hydrate was shown to have axonal protective effects in TNF-induced optic nerve degeneration. The α2-adrenoreceptor agonist brimonidine was also shown to exert axonal protection. The current study aimed to elucidate whether additive axonal protection was achieved by the simultaneous injection of ripasudil and brimonidine and examine the association with AMPK activation.

**Methods:**

Intravitreal administration was performed in the following groups: PBS, TNF, or TNF with ripasudil, with brimonidine, or with a combination of ripasudil and brimonidine. Axon numbers were counted to evaluate the effects against axon loss. Immunoblot analysis was performed to examine phosphorylated AMPK expression in optic nerves, and immunohistochemical analysis was performed to evaluate the expression levels of p-AMPK and neurofilament in the optic nerve.

**Results:**

Both ripasudil alone or brimonidine alone resulted in significant neuroprotection against TNF-induced axon loss. The combination of ripasudil and brimonidine showed additive protective effects. Combined ripasudil and brimonidine plus TNF significantly upregulated p-AMPK levels in the optic nerve compared with the TNF groups. Immunohistochemical analysis revealed that p-AMPK is present in axons and enhanced by combination therapy.

**Conclusion:**

The combination of ripasudil and brimonidine may have additive protective effects compared with single-agent treatment alone. These protective effects may be at least partially associated with AMPK activation.

## Introduction

Rho kinase (ROCK) inhibitors are commercially available in some countries as a glaucoma eyedrop. ROCK inhibitors reduce intraocular pressure (IOP) by expanding empty spaces in the juxtacanalicular area and increasing retinal blood flow [1]. For example, the ROCK inhibitor netarsudil acts on the conventional outflow pathway and reduces IOP in primary open-angle glaucoma (POAG) and ocular hypertension by improving trabecular outflow facility and decreasing episcleral venous pressure [2]. Another ROCK inhibitor, ripasudil hydrochloride hydrate, also reduces IOP by increasing outflow through modulation of trabecular meshwork cell behavior and regulation of permeability in Schlemm’s canal endothelial cells [3].

The glaucoma eyedrop brimonidine tartrate is an α2-adrenoreceptor agonist that reduces IOP and is widely used in the treatment of glaucoma. It was demonstrated that topical administration of brimonidine prevented retinal ganglion cell (RGC) loss in a retinal ischemia model [4]. Moreover, it was shown that brimonidine protected RGCs in a chronic ocular hypertensive rat model [5]. It was also reported that pretreatment of optic nerve axons with brimonidine resulted in a greater survival of optic axons in an ischemic optic neuropathy model [6].

We previously reported the axonal-protective effects of ripasudil in a tumor necrosis factor (TNF)-induced axonal damage model [7] and demonstrated the axonal-protective effects of brimonidine in the TNF-induced axonal damage model [8]. There have been several recent reports showing a close relationship between TNF and glaucoma [9, 10]. Those associations are supported by previous studies showing that increased expression of TNF and TNF receptors were observed in RGCs and the optic nerve head of patients with glaucoma [11] and that TNF-mediated cell death is involved in the neurodegeneration process of glaucoma [12]. The TNF intravitreal injection model showed that axonal damage precedes the reduction of RGCs [13]. Although one possible mechanism is that brimonidine suppresses increased TNF levels, thereby resulting in RGC protection in the ischemia reperfusion model [14], the mechanism of axonal protection of ripasudil and brimonidine remains to be elucidated. In the current study, we attempted to determine whether simultaneous injection of ripasudil and brimonidine modulates axonal loss in TNF-induced optic nerve degeneration and tested the association with AMPK activation.

## Materials and methods

### Animals

Eight-week-old male Wistar rats were fed a standard diet and water ad libitum and housed in cages (23 ± 1 °C; humidity 55 ± 5%; light from 6 AM to 6 PM). All experiments were approved by the Ethics Committee for Animal Experiments of St. Marianna University School of Medicine and conducted according to the ARVO Statement for the Use of Animals in Ophthalmic and Vision Research.

### TNF Injection model

Intravitreal injection was performed as described previously [13, 15]. Rats were anesthetized with an intramuscular injection of a mixture of ketamine-xylazine. Intravitreal injection of 10 ng TNF (Sigma-Aldrich Corp., St. Louis, MO, USA) was performed in the right eye and PBS injection in the contralateral left eye. The three treatment groups received TNF and 2 pmol of brimonidine, TNF and 20 pmol of ripasudil, or TNF, 2 pmol of brimonidine, and 20 pmol of ripasudil. All treatments in the three groups were administered intravitreally at a volume of 2 μl.

### Quantification of axon numbers

Axon number counting was performed in optic nerves from 28 rats. Optic nerves were isolated 2 weeks after intravitreal administration of the compounds tested, fixed, processed, and embedded in acryl resin [15]. After staining with 1% paraphenylenediamine, axon numbers were quantified in five separate areas (center and periphery in the quadrant; 5850 μm^2^ each; totaling 29,250 μm^2^ per optic nerve) using image-processing software (Aphelion, ADCIS S.A., Hérouville Saint-Clair, France).

### Immunoblot analysis

Fouty-eight rats were used for the immunoblot analysis. Optic nerves were isolated 1 week after intravitreal administration and homogenized in protein extraction buffer. After being centrifuged, the supernatant was used for protein concentration measurement and processed in sample buffer (Bio-Rad Laboratories). Equal amounts of each sample were applied to SDS-PAGE gels (Bio-Rad Laboratories), ran, and transferred to an enhanced chemiluminescent membrane. Primary antibodies used were anti-p-AMPK (1:200, Thr172; Sigma-Aldrich), anti-AMPK (1:200; Proteintech, Rosemont, IL, USA), and anti-β-actin (1:5000; Sigma-Aldrich). After washing, the membranes were reacted with peroxidase-labeled secondary antibodies (1:5000). An electrochemiluminescence (ECL) system was used for visualization of immunoblotting.

### Immunohistochemical analysis

Four rats from each group were used in immunohistochemical analysis. Eye cups were isolated 1 week after intravitreal administration, and they were put into 4% paraformaldehyde, processed, and embedded in paraffin. Sections were made transversely through the optic nerve head. Primary antibodies used were anti-p-AMPK antibody (1:100; Sigma-Aldrich) and anti-neurofilament-L antibody (1:100; Dako). After washing, the sections were reacted with FITC-labeled anti-rabbit or rhodamine-labeled anti-mouse antibodies (Cappel, Aurora, OH, USA) in the dark. Images of p-AMPK-positive fibers in the optic nerve were captured with microscopy with Q-Capture Pro 7 (QImaging, British Columbia, Canada).

### Statistical analysis

Data are expressed as mean ± SEM. Differences among groups were analyzed by 1-way ANOVA with post hoc Tukey’s Honestly Significant Difference (HSD) test. The results were considered statistically significant when *p* < 0.05. All graphs were drawn using GraphPad Prism 10 software (GraphPad Software, Boston, MA, USA).

## Results

### Effects of ripasudil and brimonidine against TNF-induced axon loss

**Fig. 1:**
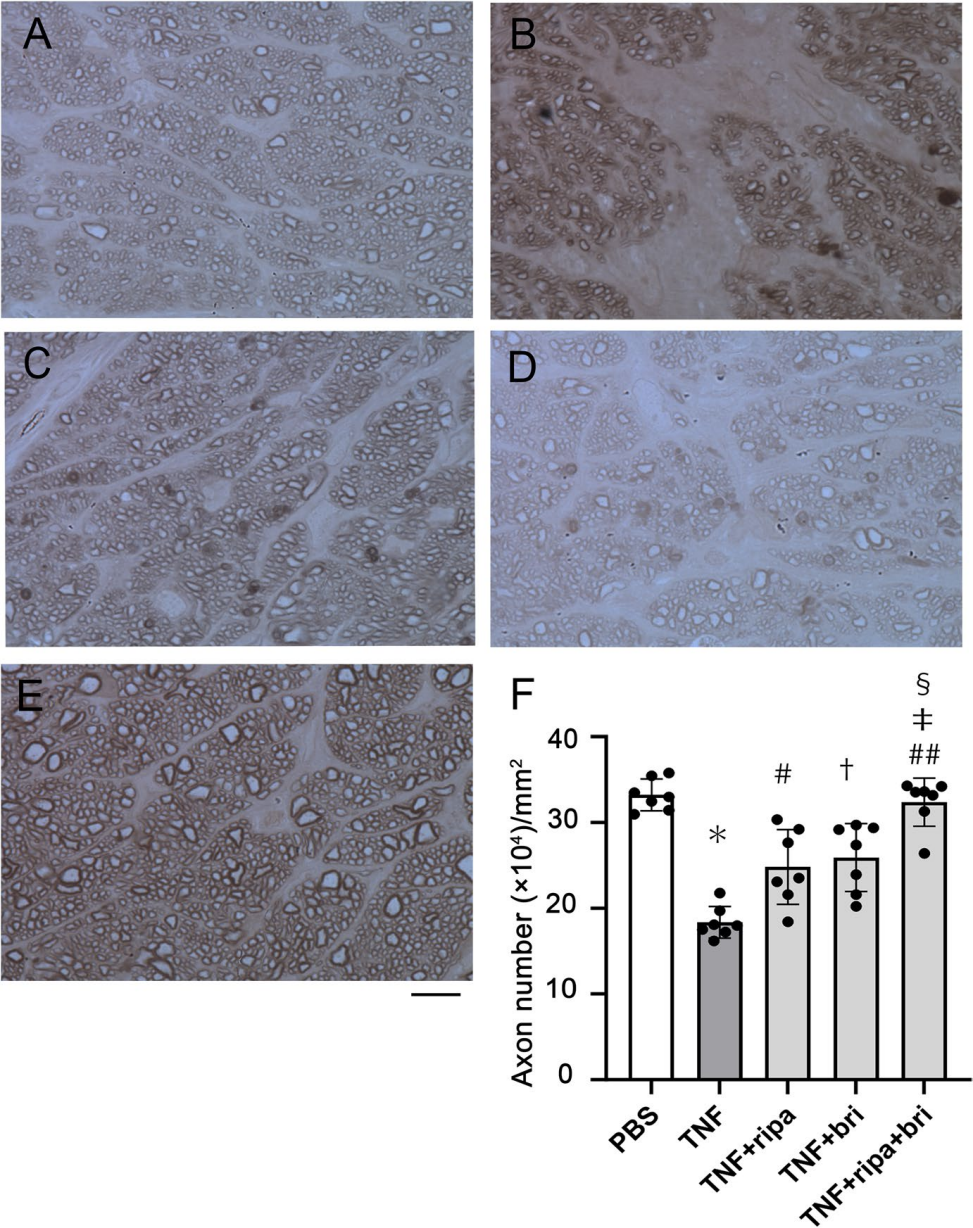
The combination of ripasudil and brimonidine exerted additive axonal effects in TNF-induced axon loss. Histologic findings 2 weeks following injection of **A** PBS, **B** TNF, **C** TNF + 20 pmol ripasudil, **D** TNF + 2 pmol brimonidine, or **E** TNF + 20 pmol ripasudil and 2 pmol brimonidine. Scale bar = 10 μm **F** Quantification of axon numbers. Each column represents mean ± SD; seven rats were used in each group except PBS group. PBS group was from contralateral left eye in the TNF group. Total 28 rats were used. ^*^p < 0.0001 compared with PBS injection; ^#^*p* < 0.005 compared with TNF injection; ^†^*p* < 0.001 compared with TNF injection; ^##^*p* < 0.0001 compared with TNF injection; ^‡^*p* < 0.001 compared with TNF + ripasudil injection; ^§^*p* < 0.01 compared with TNF + brimonidine injection

The TNF-treated group showed significant axon losses (Fig. 1B, F) compared with the control group. (control, 332,199 ± 18,575; TNF, 183,507 ± 18,504; Fig. 1A, F, *p* < 0.0001; n = 7). Compared with degenerative axons in the TNF-treated group (Fig. 1B), ripasudil (20 pmol) plus TNF showed some protective effect (TNF ripasudil, 248,223 ± 43,639; *p* = 0.0049 versus TNF alone; Fig. 1C, F; n = 7). Compared with degenerative axons in the TNF-treated group (Fig. 1B), brimonidine (2 pmol) plus TNF showed protective effects (TNF brimonidine, 259,167 ± 39,654; *p* = 0.0009 versus TNF alone; Fig. 1D, F; n = 7). Compared with degenerative axons in the TNF-treated group (Fig. 1B), the combination of ripasudil (20 pmol) and brimonidine (2 pmol) plus TNF showed significant protective effects (TNF ripasudil brimonidine, 323,605 ± 28,247; *p* < 0.0001 versus TNF alone; Fig. 1E, F; n = 7). Compared with the ripasudil (20 pmol) plus TNF group (Fig. 1C), the combination of ripasudil (20 pmol) and brimonidine (2 pmol) plus TNF showed significant protective effects (*p* = 0.0009 versus TNF + ripasudil; Fig. 1E, 1F). Compared with the brimonidine (2 pmol) plus TNF group (Fig. 1D), the combination of ripasudil (20 pmol) and brimonidine (2 pmol) plus TNF showed significant protective effects (*p* = 0.0052 versus TNF + brimonidine; Fig. 1E, F).

### Effects of ripasudil and brimonidine on p-AMPK expression

**Fig. 2:**
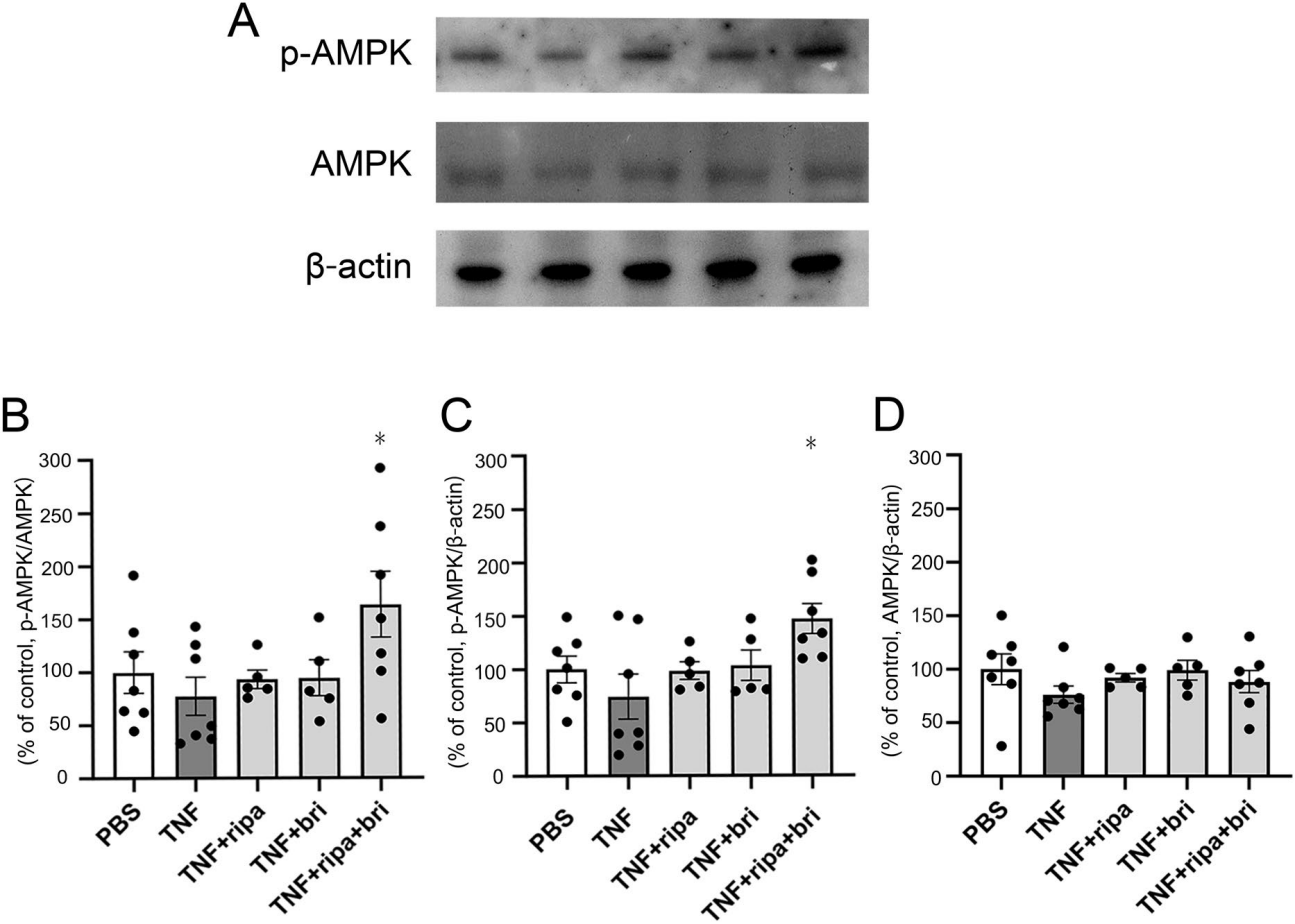
Protein expression in optic nerves 1 week following intravitreal injection. **A** p-AMPK and AMPK expressions after injection of PBS, TNF, TNF + ripasudil, TNF + brimonidine, or TNF + ripasudil + brimonidine. **B** Immunoblotting values of p-AMPK are normalized to AMPK. **C** Immunoblotting values of p-AMPK are normalized to β-actin. **D** Immunoblotting values of AMPK are normalized to β-actin. Values are expressed as percentages of control and represent mean ± SEM n = 5–7 *p < 0.05 compared with TNF injection alone

Compared with the control group, the p-AMPK level tended to decrease in the TNF group, although the difference was not statistically significant. Compared with the TNF group, p-AMPK levels in the ripasudil + TNF and brimonidine + TNF groups tended to increase, but not significantly. However, the combination of ripasudil and brimonidine plus TNF led to a significant upregulation of p-AMPK protein levels compared with the TNF-alone group (Fig. 2B, *p* = 0.0482; Fig. 2C, *p* = 0.0141). The AMPK level tended to decrease in the TNF group, but the difference was not significant (Fig. 2D).

### Localization of p-AMPK in the optic nerve

**Fig. 3:**
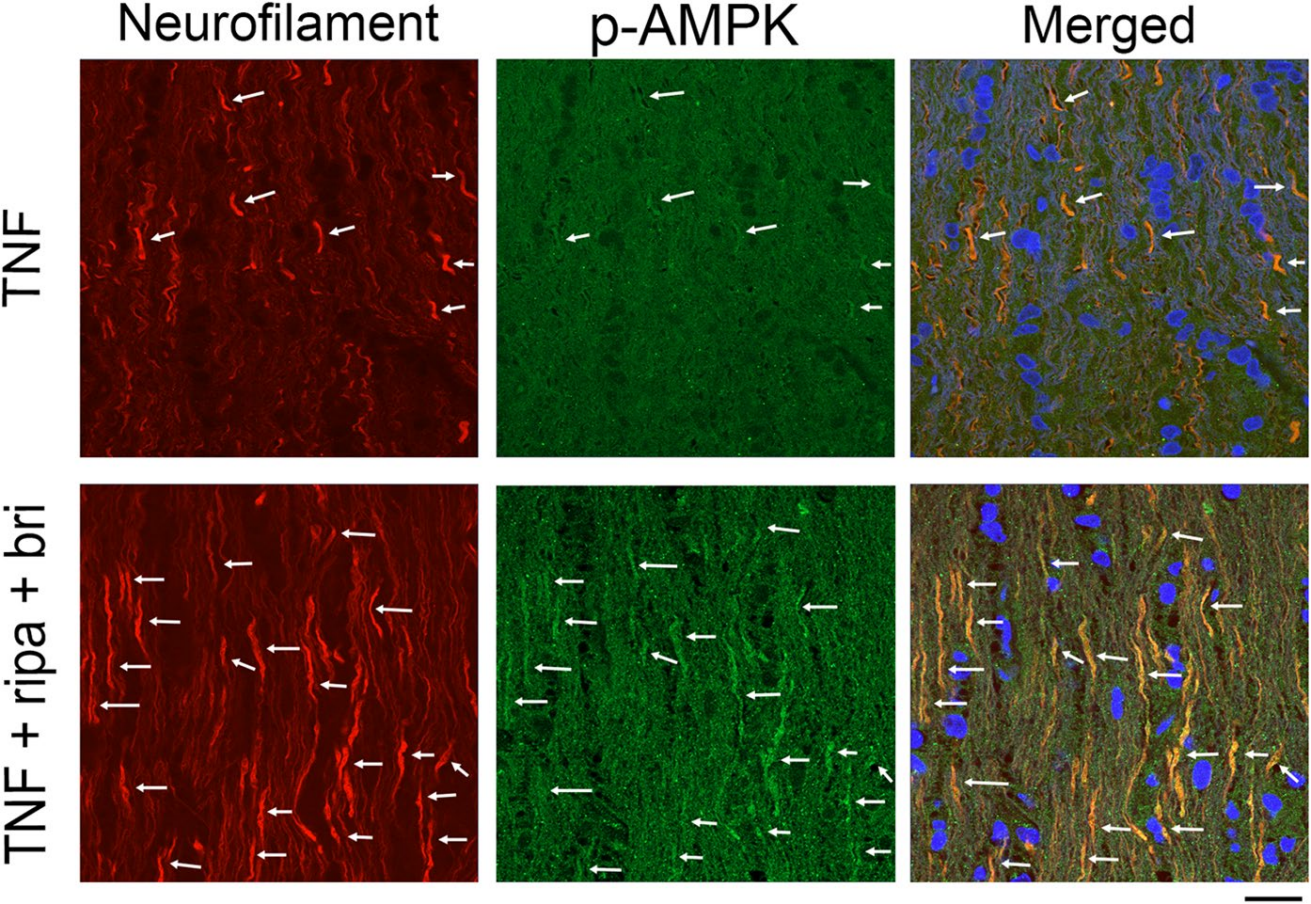
Immunohistochemical analysis of the optic nerve 1 week after injection. The p-AMPK immunoreactivity was colocalized with neurofilament-positive fibers in TNF-treated optic nerves (arrows). Abundant p-AMPK immunoreactivity was found with neurofilament-positive fibers in the TNF + ripasudil + brimonidine treatment group (arrows). Scale bar = 50 µm; n = 4 per experimental group

Modest p-AMPK immunoreactivity was found in neurofilament-positive fibers in the TNF group (Fig. 3). These p-AMPK immunopositive fibers were more obvious after combination ripasudil (20 pmol) and brimonidine (2 pmol) plus TNF treatment, and they were colocalized with neurofilament-positive fibers (Fig. 3).

## Discussion

The present study showed substantial protective effects of combined ripasudil and brimonidine administration against axonal damage caused by TNF. In our previous study, either 20 pmol of ripasudil alone or 2 pmol of brimonidine alone showed mild protective effects that were not statistically significant [7, 8]. Thus, we used the current doses to examine the additive effects of the two-drug combination in which the doses did not have significant (only marginal) effects. However, contrary to expectations, the present study showed significant axonal protection in both the ripasudil-alone and brimonidine-alone groups. This result may be related to the different methods that the number of samples in this study was greater compared with those in previous experiments. In the present study, the ripasudil and brimonidine combination group showed a significant protective effect compared with the results in the single-agent alone groups. Therefore, it is possible that ripasudil and brimonidine may exert additive protective effects. However, the caution may be needed to interpret the protective effect, because functional effects on the retina and optic nerve evaluation using the electrophysiology, pupil light reflex testing or optokinetic responses were not performed in the present study. Thus, it is unclear whether any observed protection of axons will have real functional consequences in terms of the functional preservation.

Several studies using different ROCK inhibitors focused on axonal regeneration after optic nerve injury. These ROCK inhibitors which have axonal regeneration effects include Y-27632, Y-39983, netarsudil, and ripasudil [16–19]. Among them, it was shown that ripasudil suppressed the phosphorylation of cofilin, a depolymerizing protein [19]. It was also shown that brimonidine exerted axonal regeneration through Erk phosphorylation [20]. Although axonal regeneration is important, we believe axonal protection is helpful in halting glaucoma progression [21]. A previous study showed that oral ripasudil administration delayed RGC death [22]. Several ROCK inhibitors were shown to have protective effects on RGCs in different damage models [16, 17, 23]. Our previous study demonstrated that ripasudil exhibited axon protection by promoting intra-axonal autophagy [7]. On the other hand, a previous study by Lambert et al. [24] found that continuous treatment with subcutaneous brimonidine injections significantly improved the survival of RGCs exposed to elevated intraocular pressure. We also found that brimonidine suppressed the increase in p62/SQSTM1 in axonal damage caused by TNF [8]. Since increased p62 means impairment of autophagy flux, it is possible that brimonidine promotes autophagic flux, thereby leading to axonal protection [8].

In the present study, the p-AMPK level tended to decrease in the TNF group, although the difference was not statistically significant. A recent study has shown that the expression of p-AMPK was downregulated in mouse neuroblastoma N2a cells after TNF treatment [25], suggesting dephosphorylation of AMPK by TNF. However, we can not exclude the possibility that TNF may downregulate AMPK protein [26]. Our immunoblot analysis also showed that the AMPK level tended to decrease in the TNF group without statistical significant.

Fasudil, a ROCK inhibitor, activates AMPK in skeletal muscle cells [27]. A recent study has demonstrated that ripasudil upregulates p-AMPK in bovine corneal endothelial cells [28]. We have recently found that the ROCK inhibitor netarsudil increased p-AMPK in the optic nerve with upregulation of autophagy [29]. Very recent studies have also shown a close relationship between ROCK inhibition and AMPK activation in several different cells [30, 31], suggesting that ROCK inhibitors may act as upstream effectors of AMPK. On the other hand, the α2-adrenoreceptor agonist dexmedetomidine protected cardiovascular endothelial cells via AMPK-autophagy [32]. In addition, it was shown that dexmedetomidine exerted neuroprotection via AMPK-autophagy in SH-SY5Y-APP cells [33]. These findings are in line with the present findings that the combination of ripasudil and brimonidine activates AMPK in the optic nerve. Treatment with the AMPK activator A769662 increased Thr-172 phosphorylation of AMPK, resulting in stimulated PGC-1α-directed mitochondrial biogenesis and autophagy induction [34]. Treatment with A769662 exerted axonal protection associated with AMPK activation and autophagy induction in TNF-induced optic nerve degeneration [29]. A very recent study showed that A769662 protected UVA-induced retinal pigmented epithelial cells [35]. Taken together, these findings suggest that the combination of ripasudil and brimonidine may have axonal-protective effects with possible involvement of AMPK activation.

## Data Availability

The datasets used and/or analyzed during the present study are available from the corresponding author on reasonable request.
